# Prostate-specific IL-6 transgene autonomously induce prostate neoplasm through amplifying inflammation in the prostate and peri-prostatic adipose tissue

**DOI:** 10.1186/s13045-016-0386-7

**Published:** 2017-01-11

**Authors:** Gang Liu, Jinyu Zhang, Lewis Frey, Xiao Gang, Kongming Wu, Qian Liu, Michael Lilly, Jennifer Wu

**Affiliations:** 1 Department of Medicine, University of Washington, Seattle, WA USA; 2 Department of Microbiology and Immunology, Medical University of South Carolina, Charleston, SC 29425 USA; 3 Public Health Science, Medical University of South Carolina, Charleston, SC 29425 USA; 4 Department of Oncology, Tongji Medical College, Huazhong University of Science and Technology and Tongji Hospital, Wuhan, China; 5 Department of Hematology and Oncology, Medical University of South Carolina, Charleston, SC 29425 USA; 6 Present address: Department of Laboratory Medicine, The Third Hospital of South Medical University, Guangzhou, China; 7 Hollings Cancer Center, Medical University of South Carolina, Charleston, SC 29425 USA

**Keywords:** IL-6, Prostate neoplasm, Transgenic mouse, Inflammation

## Abstract

**Background:**

The causative role of the pro-inflammatory cytokine IL-6 in prostate cancer progression has been well established at molecular level. However, whether and how IL-6 may play a role in prostate cancer risk and development is not well defined. One limitation factor to acquiring this knowledge is the lack of appropriate animal models.

**Methods:**

We generated a novel line of prostate-specific IL-6 transgenic mouse model. We compared the prostate pathology, tumorigenic signaling components, and prostate tumor microenvironment of the IL-6 transgenic mice with wild type littermates.

**Results:**

With this model, we demonstrate that IL-6 induces prostate neoplasm autonomously. We further demonstrate that transgenic expression of IL-6 in the prostate activates oncogenic pathways, induces autocrine IL-6 secretion and steadily-state of STAT3 activation in the prostate tissue, upregulates paracrine insulin-like growth factor (IGF) signaling axis, reprograms prostate oncogenic gene expression, and more intriguingly, amplifies inflammation in the prostate and peri-prostatic adipose tissue.

**Conclusions:**

The pro-inflammatory IL-6 is autonomous oncogene for the prostate. IL-6 induces prostate oncogenesis through amplifying local inflammation. We also presented a valuable animal model to study inflammation and prostate cancer development.

**Electronic supplementary material:**

The online version of this article (doi:10.1186/s13045-016-0386-7) contains supplementary material, which is available to authorized users.

## Background

Emerging evidence indicated that the pro-inflammatory cytokine IL-6 may play a causative role in prostate cancer progression [1]. For instance, IL-6 has been shown to facilitate prostate cancer progression to androgen-independent disease and potentially to promote bone metastasis and neuroendocrine differentiation (NED) [2–7]. Elevation of serum levels of IL-6 or activation of IL-6 signaling pathways in the tumor tissue correlates with the shortened overall survival and time to progression in prostate cancer [8–13]. In vitro and xenograft in vivo studies have demonstrated that IL-6 plays a causative role to promote oncogene-immortalized non-tumorigenic prostate epithelial cells to overt malignancy [14]. Conclusions from recent clinical studies propose that serum IL-6 can be a negative prognostic biomarker for prostate cancer [10].

IL-6, a multi-functional cytokine that can be produced by various cell types, including immune/inflammatory cells (monocytes, macrophages, B cells, T cells, nature killer cells), fibroblasts, keratinocytes, endothelial cells, and also tumor cells, plays a pivotal role in controlling cell differentiation and cancer cell survival [15, 16]. IL-6 signals through the adaptor molecule gp130 via canonical membrane bound IL-6R and/or alternatively soluble IL-6R trans-signaling in IL-6R-gp130^+^ cells to initiate the downstream activation cascade [17, 18]. It has been well established that activation of the signal transduction and activator of transcription 3 (STAT3), a key effector protein of IL-6 signaling, is critical in initiating oncogene transcription and cancer progression [19]. IL-6 can promote tumorigenic conversion of oncogene-immortalized benign cells through STAT3-mediated trans-activating other cellular signaling pathways, such as MAPK, PI3K/AKT, and insulin-like growth factor I receptor (IGF-IR) signaling axis [14, 20]. Persistent activation of STAT3 in prostate carcinomas has been correlated with the shortened survival of cancer patients [8–12]. Upregulation of IL-6 and activation of STAT3 autocrine pathway have been shown to account for the major mechanisms of cancer cell resistance to therapy [19, 21, 22]. Based on these understandings, recent studies have been focused on targeting IL-6 or IL-6 signaling pathways in cancer patients [19, 23–25].

Although the significance of IL-6 in prostate cancer progression is well established, whether IL-6 plays a causative role in prostate cancer risk and early development is not clearly well defined. Polymorphisms of IL-6 gene have been associated with prostate cancer risk [26–28]; however, *bona fida* evidence of IL-6 as a sole factor in prostate cancer risk is lacking. Most of pro-tumorigenic evidence of IL-6 in prostate cancer was achieved from experiments of oncogene-immortalized cell lines or clinical correlation studies. To address the fundamental biological question whether IL-6, as the major pro-inflammatory cytokine, can initiate prostate tumorigenesis in an autologous state, here, we developed a prostate-specific IL-6 transgenic mouse line in which IL-6 was directed to express specifically in the prostate by the rat probasin promoter. With this model, we demonstrated that overexpression of IL-6 alone was sufficient to induce prostate epithelium malignant neoplasm. We demonstrated that enforced expression of IL-6 in the prostate activated STAT3 pathway in the epithelium and stroma, induced an IL-6 autocrine and insulin-like growth factor (IGF) paracrine loop, reprogrammed prostate oncogenic gene expression, and amplified pro-tumorigenic inflammation in the prostate tissue microenvironment and peri-prostatic adipose tissue. Our study suggests that IL-6 is an unconventional “oncogene” for the prostate. Moreover, our prostate-specific IL-6 transgenic mouse can serve as a valuable model to study inflammation-associated prostate cancer prevention.

## Results

### IL-6 transgene induces early prostate neoplastic transformation

**Fig. 1:**
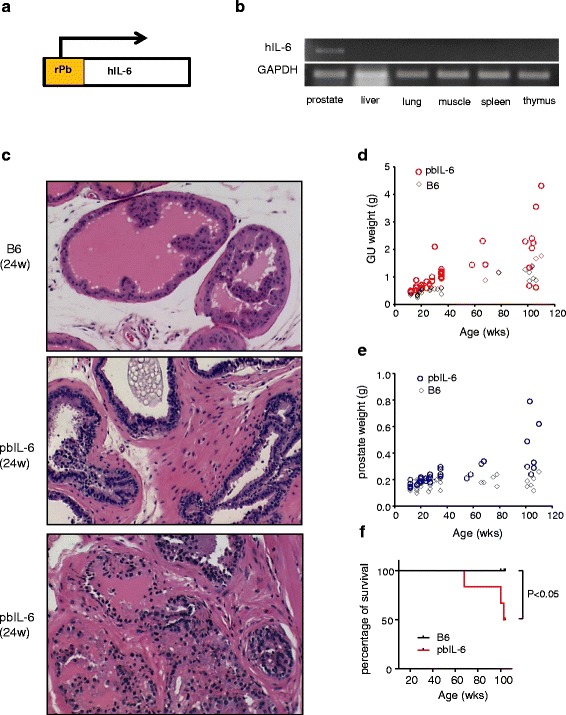
Prostate histology and weight comparison between pbIL-6 mice and B6 WT mice. **a** Diagram of rPB-hIL-6 (human IL-6) expression cassette construction which was microinjected into fertilized C57BL/6 embryos to generate B6.PbIL-6 transgenic mice. **b** RT-PCR showed the hIL-6 was expressed specially in the prostate tissue in the transgenic mice. **c** Representative histology comparison of prostate from pbIL-6 mice and B6 WT littermates at 24 weeks of age. **d**, **e** Genitourinary (GU) and prostate weight comparison of pbIL-6 mice and B6 WT. **f** Keplan-Meier curve of overall survival by 104-week old

Previously, we described that IL-6 facilitated prostate tumorigenesis and progression through autocrine IL-6 loop and re-programming oncogenic transcriptional profiles using oncogene-immortalized benign prostate epithelial cell lines [14]. Inspired by these findings, in this study, we aimed to address whether IL-6 has a causative role in de novo spontaneous prostate tumor initiation and thus generated prostate-specific IL-6 transgenic mouse lines. The encoding transgene of human IL-6 (hIL-6), which has been shown to activate downstream signaling cascade similarly in both human and mouse cells [29, 30], was expressed in the prostate of the C57BL/6 (B6) mice directed by the rat probasin (rPb) promoter [31] (Fig. 1a). We chose to express human IL-6 so that we could differentiate the expression of hIL-6 transgene from the autocrine expression of mouse IL-6 (mIL-6). The founder line that expressed the transgene IL-6 in the prostate was designated as pbIL-6. Integration of a single copy of an intact transgene was confirmed by genomic PCR against a limited template dilution standard (data not shown). Prostate-specific expression of the transgene hIL-6 was detected by RT-PCR (Fig. 1b).

**Fig. 2:**
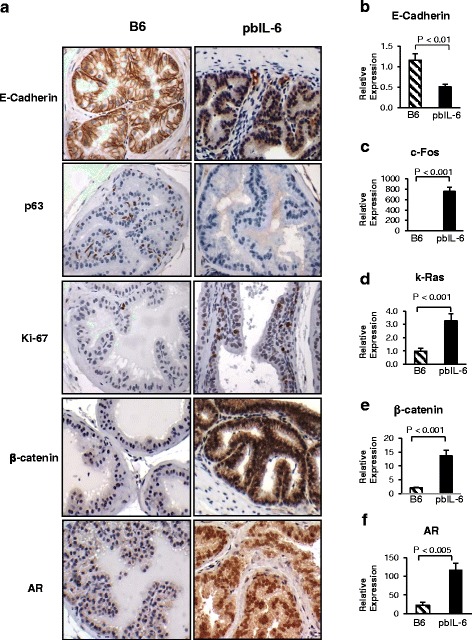
Expression of neoplasm related markers in the prostate of pbIL-6 mice and WT littermates. **a** Immunohistochemistry staining of panel of neoplasm related markers. **b**–**f** Relative expression levels of neoplasm related markers assessed by quantitative RT-PCR. Data shown were representative images and expression data of prostates from 24-week-old pbIL-6 mice and B6 WT littermates. *AR* androgen receptor

To demonstrate the consequences of prostate-specific expression of hIL-6 in the adult prostate, we compared the histological characteristics of pbIL-6 transgenic mice with the wild type B57CL/6 (B6) littermates. The prostate glands from pbIL-6 transgenic mice uniformly exhibited varying degrees of prostate intraepithelial neoplasia (PIN)-like lesions at the age of 16-week old as we have examined, which were not observed in the B6 WT littermates (data not shown). By 24 weeks of age, neoplasia was evident in the pbIL-6 prostates. Specifically, the prostate exhibited increased epithelial tufting, overlapping of cells, enlarged nuclei of variable sizes and shapes with prominent nucleoli, and thickening of the stroma (Fig. 1c). As animals age, prostate glands in the pbIL-6 mice more predominantly exhibited carcinoma-like feature, such as increased proliferation, enlarged nucleoli, and disappearance of basal cells (Table 1 and Fig. 2). In two out of ten examined aged mouse (>100-week old), prostate carcinoma progressed to poorly differentiated (Additional file 1: Figure S1). Consistent with the progressive neoplasia development in pbIL-6 mice as animal ages, the rate of weight increase of prostate and genitourinary (GU) by age was significantly greater in pbIL-6 mice than in B6 WT littermates (Fig. 1d, e). Notably, unlike human, wild type mice only develop prostate intraepithelial neoplasia (PIN) but not spontaneous prostate carcinoma as they age [32]. Overall, the pbIL-6 animals presented higher mortality due to progressive tumor development in the prostate, with rare incidence in the liver (Fig. 1f).

**Table 1:** Summary of the prostate pathology of pbIl-6 mice and wild type littermate surveyed at various ages

Age (weeks)	16–20	24–28	52–72	>100
pbIL-6				
PIN	100%	100%	100%	100%
	(13/13)	(11/11)	(16/16)	(10/10)
Tumor (WD)	100%	100%	100%	100%
	(13/13)	(11/11)	(16/16)	(10/10)
Tumor (PD)	0/13	0/11	6.2%	20%
			(1/16)	(2/10)
B6 (WT)				
PIN	0/20	4.5%	8.3%	12.5%
		(1/22)	(1/12)	(1/8)
Tumor (WD)	0/20	0/22	0/22	0/8
Tumor (PD)	0/20	0/22	0/22	0/8

*PIN* prostate intraepithelial neoplasia, *WD* well-differentiated tumor, *PD* poorly differentiated tumor

In our previous report, we described multiple effects of IL-6 on immortalized benign human prostate epithelial cells [14]. Consistent with these findings, the prostate epithelium of the transgenic pbIL-6 mice demonstrated a focally dramatic decrease of membranous expression of E-cadherin (Fig. 2a, b). The loss of E-cadherin signifies a reduction in cell-cell adhesion and a shift of the prostate luminal cells from a clearly differentiated epithelial to a more mesenchymal phenotype and also an early stage of transformation [33, 34]. The disrupted expression of basal cell marker p63 in the prostate gland from the pbIL-6 mice also signifies the early transformation events of the prostate [35, 36] (Fig. 2a). Consistent with our previous report of the tumorigenic potential of IL-6 in epithelial cell lines [14], the prostate epithelium from pbIL-6 mice had a significant higher index of Ki-67 positivity (Fig. 2a), suggesting an active proliferation state. Moreover, in comparison to WT littermate, not only a markedly overall elevation of the oncogene β-catenin but also more significantly elevated accumulation of β-catenin in the nucleus was presented in the prostate epithelium of the pbIL-6 mice (Fig. 2a, e). Furthermore, in comparison to the B6 WT littermates, androgen receptor (AR) was not only significantly increased in the levels of expression in the prostate epithelium of the pbIL-6 mice, but also predominantly translocated to the nucleus (Fig. 2a, f), an indication of increased AR activity. Given that IL-6 has been known to increase AR activity in castration-resistant prostate cancer [37], together, these molecule features of the prostate epithelium of the pbIL-6 mice endowed its neoplasia features and tumorigenic properties, which were further substantiated by elevated expression of oncogenes, such as c-Fos and k-Ras as measured by quantitative RT-PCR (Fig. 2c, d).

### IL-6 transgene activates autocrine IL-6 loop and upregulates the IGF-I signaling axis in the prostate

**Fig. 3:**
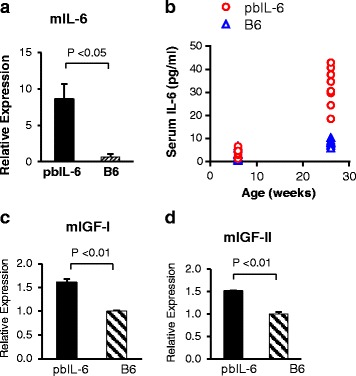
IL-6 induces autocrine IL-6 secretion and upregulates IGF signaling axis in the prostate. **a** Quantitative RT-PCR demonstrating significantly increased mouse IL-6 (mIL-6) expression in the prostate of pbIL-6 mice in comparison to B6 WT littermates. **b** Significant elevation of serum mIL-6 in pbIL-6 mice in comparison to B6 WT littermates. **c**, **d** Significant elevation of IGF-I and IGF-II expression in the prostate of pbIL-6 mice in comparison to B6 WT littermates. Data shown were representatives of mice at the age of 26-week old

In the previous report, we demonstrated that IL-6 stimulates the autocrine IL-6 loop in the immortalized benign prostate epithelial cells [14]. To investigate whether this is a *bona fida* effect of IL-6 signaling event during *de novo* tumorigenesis, we examined the expression of mouse IL-6 (mIL-6) in the prostate. Quantitative RT-PCR revealed a significant induction of mouse IL-6 expression in the prostate of pbIL-6 mice in comparison to the WT B6 littermate as we examined at 16 weeks of age (Figs. 3a, *P* < 0.01). By 24 weeks of age, significantly elevated serum levels of mIL-6 were detected in pbIL-6 mice (Figs. 3b, *P* < 0.01), presumably due to the more severe destruction of the prostate architect and release of secretory mediators. These results suggested that IL-6 in prostate tissue environment can induce *bona fida* IL-6 autocrine secretion. We also observed autocrine IL-6 induction in mouse prostate tumor cell lines exposed to exogenous IL-6 (Additional file 1: Figure S2).

We and the others have previously described that IL-6 signaling trans-activates the endocrine IGF signaling axis in cancer cells [14]. Activation of IGF signaling axis has been shown to play an essential role in tumor cell survival and proliferation [33]. In pbIL-6 mice, a significantly increased expression IGF-I and IGF-II was demonstrated by quantitative RT-PCR (Fig. 3c, d). Collectively, these data suggest that trans-activating IGF signaling axis may also contribute to IL-6-induced epithelium transformation.

### IL-6 transgene universally activates STAT3 pathway and reprograms gene expression in the prostate tissue

**Fig. 4:**
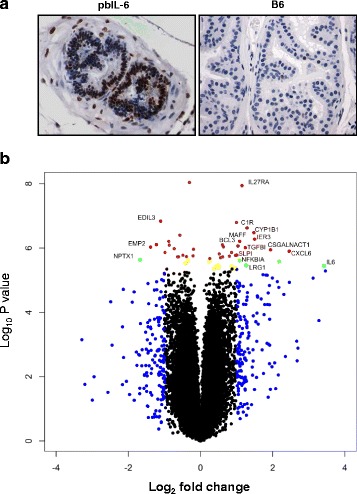
IL-6 activates STAT3 pathway and reprograms prostate gene expression to be pro-tumorigenic. **a** IHC demonstrating phosphorylation of STAT3 in the prostate epithelium and stromal components in pbIL-6 mice, whereas pSTAT3 was rarely detected in prostate from the B6 WT littermates. **b** A volcano plot demonstrating upregulation of genes associated with oncogenic and inflammation in the prostate of pbIL-6 mice. The log2 fold change of genes between four samples of IL6 mice vs. two WT littermates is plotted against the negative log10 *p* value in the volcano plot. The *brown* and *red dots* are above bonferroni adjusted *p* values for 22,623 tests for alpha 0.05. The *yellow* and *green dots* are genes above an alpha of 0.1 and below 0.05. The *red, green* and *blue dots* are genes with average fold greater than 1 for the log2 ratio. The *green* and *red* are above the log2 ratio of 1 and are significant at the 0.1 or 0.05 alpha level, respectively. The function of the most upregulated genes are indicated in Additional file 1: Table S1-4

Engagement of IL-6/IL-6Rα or IL-6/sIL-6Rα complex to the signaling adaptor molecule gp130 triggers the activation of downstream signaling cascade, most prominently the JAK2/STAT3 pathway [38]. Phosphorylation of STAT3 and translocation to the nucleus are the critical events for initiating the oncogenic transcriptional activity [39]. As shown in Fig. 4a, the prostate of pbIL-6 mice exhibited high levels of phosphorylated STAT3 (pSTAT3), whereas the prostate from WT littermates rarely presented positivity for pSTAT3. Intriguingly, pSTAT3 was present not only in the prostate epithelium cells but also in the stromal components of pbIL-6 mice. These data suggest that IL-6 signaling may pose effects on the prostate epithelium and stromal environment to induce neoplastic transformation.

We further addressed how expression of IL-6 in the prostate may impact prostate gene expression profiles using Affymetrix whole-transcript RNA array analyses. One hundred thirty genes were identified at least 2-fold upregulated in the prostate of pbIL-6 mice in comparison to the WT littermates at the significant level of *P* < 0.05. The most significantly regulated genes are indicated in the volcano plot of Fig. 4b and Additional file 1: Tables S1–S4. Notably, a large portion of these upregulated genes are associated with inflammation, oncogenesis, or metabolism. The most relevant upregulated genes, such as cytokine IL-6 and oncogenes c-JUN, β-catenin, and Ras have been validated by quantitative RT-PCR (Figs. 2c–e and 3a). IGF-I and the androgen signaling downstream gene TMPRSS2 were also shown in the gene expression array to be upregulated (Additional file 1: Table S3). Many of these transcriptional changes are consistent with our previous findings with in vitro IL-6 overexpression studies [14]. Together, these data indicate that expression IL-6 not only intrinsically reprograms the prostate to express pro-tumorigenic genes but also primes a pro-inflammatory tissue microenvironment whereby the combinatory effect may contribute to prostate neoplasm.

### IL-6 amplifies pro-tumorigenic inflammation in prostate tissue microenvironment

Emerging evidence suggests that chronic or recurrent inflammation may initiate and promote cancer development, including prostate cancer [40–43]. This process involves multiple inflammatory cells as well as a broad array of inflammatory cytokines [44]. IL-6, as a major pro-inflammatory cytokine can be secreted by an array of inflammatory cell types, are not only the growth factor for epithelial cell, but also critical for the survival and proliferation of inflammatory cell types in the tissue microenvironment. This process is frequently referred as “smoldering” inflammation [40, 44–46].

**Fig. 5:**
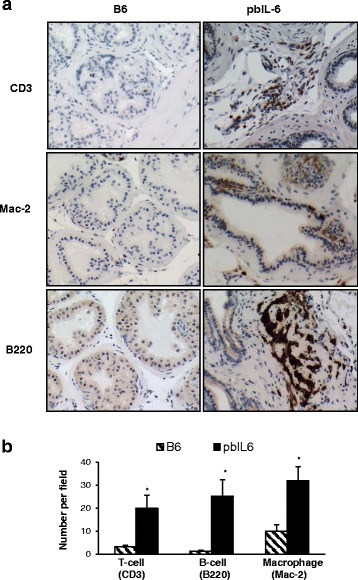
IL-6 amplifies inflammation in the prostate tissue. **a** Representative micrographs of IHC demonstrating increased infiltration of T lymphocytes (CD3^+^), macrophage (MAC-2^+^), and B lymphocytes (B220^+^) in prostate tissue of pbIL-6 mice in comparison to B6 WT littermates. **b** Quantitative scoring of each inflammation cell types in the prostates of pbIL-6 mice and B6 WT littermates. Ten random fields containing prostate glands and stroma of each prostate section were scored. Prostates from 6 to 10 animals from each group were evaluated. *P < 0.05

**Fig. 6:**
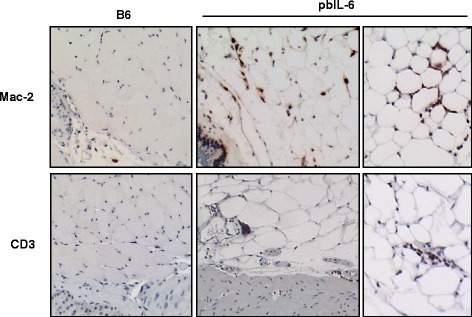
IL-6 induces inflammation in the peri-prostatic adipose tissue. Representative micrographs of IHC demonstrating increased infiltration of macrophage (MAC-2^+^) and T lymphocytes (CD3^+^) in the peri-prostatic adipose tissue of pbIL-6 mice

In general, an enriched infiltration of inflammatory cells was present in the prostate of the pbIL6 mice (data not shown). We thus characterized these inflammatory cell types by immunohistochemistry staining (IHC) with specific markers. The enriched infiltration of T lymphocytes (CD3^+^), macrophages (MAC-2^+^), and B lymphocytes (B220^+^) was remarkable and significantly increased in the prostate of pbIL6 mice in comparison to WT littermates (Fig. 5a, b), all of which are known to be IL-6- producing cells. Intriguingly, a rich infiltration of macrophages and T lymphocytes was also evident in the peri-prostatic adipose tissue in the pbIL6 mice, whereas infiltration is rare in the peri-prostatic adipose tissue of B6 WT littermates (Fig. 6). Together, these data suggest that enriched IL-6 expression in the prostate can amplify the inflammatory responses in the local tissue and peri-prostatic adipose tissue, which may confer as one of the pathways to induce neoplasm in the prostate.

## Discussion

The link between IL-6 and prostate cancer progression has been well established [1]. However, whether IL-6 alone can induce *de novo *prostate tumor initiation in an autologous state is unknown. Using the novel prostate-specific IL-6 transgenic mice, we clearly demonstrate the oncogenic property of IL-6. We show that elevated expression of IL-6 alone in the prostate is sufficient to induce local neoplasm. We further show that constitutive IL-6 expression in the prostate, resembling chronic inflammation, activates STAT3, reprograms prostate gene transcription to pro-tumorigenic, activates the autocrine IL-6 and paracrine IGF signaling axis, and amplifies inflammation in the prostate and peri-prostatic adipose tissue. This is the first study, to our knowledge, that established a direct link between IL-6 and de novo tumorigenesis in the prostate. Our data conclude that IL-6 is an “unconventional” oncogene in prostate tumorigenesis.

Given that IL-6 is a common cytokine produced by many inflammatory cell types, our study also suggests a direct link between inflammation and carcinogenesis. The link between inflammation and cancer has been suggested since centuries ago by Dr. Rudolf Virchow [45]. However, the conventional wisdom considered inflammatory acts as a “stimuli” to other genotoxic events to facilitate cancer development. Our current study clearly demonstrated that inflammation can also act as an “ignitor” to autonomously initiate de novo tumor development without other genomic insults, in which context the pro-inflammatory cytokine IL-6 serves as a critical mediator or “lynchpin” [39].

**Fig. 7:**
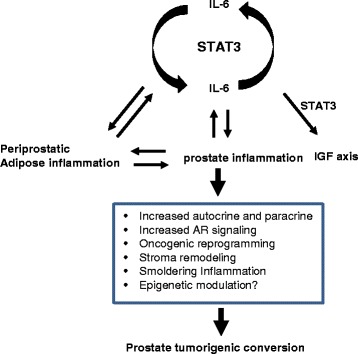
Proposed mechanism of the oncogenic property of IL-6 in the prostate. The pro-inflammatory cytokine IL-6 in the prostate, as a result of inflammation or peri-prostatic obesity, induce autocrine and paracrine pathway and amplify inflammation in the prostate tissue environment and peri-prostate adipose tissue which subsequently induces transcriptional reprogramming in the prostate and tumorigenesis

Our prostate-specific IL-6 transgenic mice presented several intriguingly features in the prostate: (1) activation of STAT3 in the stroma; (2) enriched infiltration of inflammatory cell types in the prostate; and (3) enriched infiltration of inflammatory cells in the peri-prostatic adipose tissue. IL-6 is the most well-known conventional activator of STAT3 [19, 47]. In normal cells, activation of STAT3 by IL-6 is regulated by a negative feedback loop through the activity of suppressor of the cytokine signaling 3 (SOCS3) whose expression can be induced by IL-6 signaling [48, 49]. SOCS3 can bind to the Y759 residue on the adaptor molecule gp130 and thus preventing constitutive IL-6 signaling and activation of STAT3 in a steadily state [48, 49]. When cells are insulted by abnormal physiological conditions, STAT3 can be constitutively activated by IL-6 signaling due to functional silencing of the SOCS3 gene through epigenetic hypermethylation [49, 50]. Prostate cancer patients who have methylation in the promoter region of SOCS3 presented a more aggressive phenotype [51]. As inflammation is known to cause epigenetic perturbation [52, 53], cancers in this subset of patients are thus suggested to be inflammation-originated. Although not determined in this study, our findings warrant a future investigation on epigenetic modifications in the prostate. Given the current understanding that activation of STAT3 may also be the “hub” of the interplay among adipose tissue, inflammation, and cancer [39, 54], our observations suggest that IL-6 may exert its “oncogenic” property through multiple pathways (Fig. 7).

Elevation in IL-6 expression and loss of E-cadherin have been linked with increase cancer stem cell population in various cancer types [55–57]. In this study, we did not observe positivity for cancer stem cells with markers such as CD133, OCT4, or Nanog (data not shown), suggesting that rising or re-populating of cancer stem cells is not the major mechanism by which IL-6 induces prostate tumorigenesis in current model.

## Conclusion

Prostate cancer is a heterogeneous neoplasm which is regulated by factors, such as age, hormones, obesity, and dietary habits, in addition to genomic insults common to other cancers. Many epigenetic and clinical follow-up studies have suggested a strong link between chronic inflammation and prostate cancer risk [43, 58–60]. However, to date, our understandings are limited by lacking appropriate animal models to study the development of prostate cancer from chronic inflammation. In the current study, we not only demonstrated that IL-6 is an oncogene for prostate cancer, but also present a valuable model that recapitulates the role of inflammation in prostate cancer development.

## Methods

### Generation of transgenic mice

Mice were bred and housed under specific pathogen-free conditions in the University of Washington animal facility in accordance with the institutional guidelines. All mice used in this study were on the C57BL/6 (B6) background. The rPB-IL6 expression cassette was constructed by replacing the SV40T human IL-6. The entire rPB-IL6 expression cassette was gel isolated following digestion with Hind III and was microinjected into fertilized B6 embryos at University of Washington Comparative Medicine transgenic core facility. Transgenic progeny were identified by PCR analysis of DNA extracted from tail biopsies using the forward primer specific for rPB (5′-acaagtgcatttagcctctccagta-3′) and the reverse primer specific for IL-6 (5′-tgtgtcttggtcttcatggc-3′). All experimental mice were randomly assigned to cohorts and euthanized at indicated age for evaluation of GU and the prostate.

### Quantitative RT-PCR

Total RNA was extracted using TRizol (Invitrogen) followed by treatment with DNAase I (Fermentas) to exclude the genomic DNA before reversal transcription. Complementary DNA (cDNA) was synthesized using the SuperScript II kit (Invitrogen). A volume of 1 μL of cDNA was mixed with Power SYBR Green PCR MasterMix (Applied Biosystem, Carlsbad, CA, USA), and specific primer sets were added to a final concentration of 400 nM in 20 μl of reaction mixture. The reaction was performed on an ABI9700 Machine. Data were analyzed using the Lightcycler software v3.5 (Roche Applied Science, Indianapolis, IN, USA). Each sample was assayed in triplicates. Target mRNA levels were normalized against mouse GAPDH. The primers used are listed in Additional file 1: Table S5.

### Microarray analyses

Total RNA of the prostates from four 24-week-old pbIL-6 transgenic male mice and wild type C57BL/6 littermates was obtained as described above. After RNA quality confirmation with a Bioanalyzer (Agilent), 300 ng of each RNA sample was used in the Affymetrix Whole-Transcript Sense Target Labeling Assay (Rev 3), followed by hybridization to a GeneChip Mouse Gene 1.0 ST Array. Eight GeneChips were used to provide biological replicates of each genotype. The Affymetrix Expression Console (v 1.1) was used to normalize data and determine signal intensity (RMA-Sketch). Analysis was performed using DAVID Bioinformatic software and R2 statistical software with bonferroni correction.

### Histological and immunohistochemical examination

The mouse prostate tissues were fixed in 10% formaldehyde and embedded in paraffin wax. Five-micrometer sections were cut and stained with H&E for pathological evaluation. Sections were also stained with antibodies specific for: (1) E-cadherin (Santa Cruz); (2) p63 (Thermo Scientific); (3) Ki67 (Thermo Scientific); (4) β-catenin antibody (AbCAM); (5) AR (Santa Cruz); (6) pSTAT3 (Cell signaling); (7) macrophage (F4/80 or Mac-2; eBioscience); (8) B cells (B220, eBioscience); and (9) anti-CD3 (Thermo Scientific). The staining procedure has been previously described [14]. Briefly, sections were deparaffinized and incubated for 10 min in 10 mM citrate buffer (pH 6.0) at 95 °C for antigen retrieval. Endogenous peroxidase activity was quenched with 3% hydrogen peroxide in methanol. After quenching endogenous peroxidase activity and blocking nonspecific binding, slides were incubated with specific primary antibody overnight at 4 °C followed by subsequent incubation with the appropriate biotinylated secondary antibody provided with Vectastain Elite ABC Kit. Color was developed with DAB as the perioxidase substract. All slides were counterstained with hematoxylin and mounted with Permount. Ten randomly selected fields of IHC-stained sections of the prostates from individual mice were counted for the positively stained cells and used for statistical analysis.

### Statistical analysis

All results are expressed as the mean ± SEM. Differences between the mean of groups were analyzed using student’s *t* test with one-way ANOVA analyses. In most cases, *P* < 0.05 was considered as significant.

## Additional file

Additional file 1: Figure S1. Representative histology of a 103-week-old mouse (#1825) that developed poorly differentiated tumor in the prostate. CD45 stains for all leukocyte antigens in the infiltrates. Supplement Figure 2. Exogenous human IL-6 induces paracrine mouse IL-6 expression in two mouse prostate tumor cell lines, the TRAMP-C2 and MyC-Cap. Fifty nanogram per milliliter of human IL-6 was supplemented in the culture media. Cells were harvested at 24 and 48 hr of culture. Relative mouse IL-6 expression was assessed by quantitative RT-PCR. Table S1. Function of the most significantly upregulated genes with more than log2 fold changes in the volcano plot. Table S2. Upregulated inflammation-associated genes in the prostate of pbIL-6 mice that are not highlighted on the volcano plot. Table S3. Upregulated oncogenic genes in the prostate of pbIL-6 mice that are not highlighted on the volcano plot. Table S4. Upregulated metabolism-associated genes in the prostate of pbIL-6 mice that are not highlighted on the volcano plot. Table S5. List of primers used for qRT-PCR. (PDF 329 kb)

## References

[1] Culig Z (2014). Proinflammatory cytokine interleukin-6 in prostate carcinogenesis. Am J Clin Exp Urol.

[2] Ara T, Declerck YA (2010). Interleukin-6 in bone metastasis and cancer progression. Eur J Cancer.

[3] Corcoran NM, Costello AJ (2003). Interleukin-6: minor player or starring role in the development of hormone-refractory prostate cancer?. BJU Int.

[4] Ishiguro H (2009). aPKClambda/iota promotes growth of prostate cancer cells in an autocrine manner through transcriptional activation of interleukin-6. Proc Natl Acad Sci U S A.

[5] Paule B (2007). The NF-kappaB/IL-6 pathway in metastatic androgen-independent prostate cancer: new therapeutic approaches?. World J Urol.

[6] Santer FR (2010). Interleukin-6 trans-signalling differentially regulates proliferation, migration, adhesion and maspin expression in human prostate cancer cells. Endocr Relat Cancer.

[7] Zhu Y (2014). Interleukin-6 induces neuroendocrine differentiation (NED) through suppression of RE-1 silencing transcription factor (REST). Prostate.

[8] Chen MF (2013). IL-6 expression regulates tumorigenicity and correlates with prognosis in bladder cancer. PLoS One.

[9] Knupfer H, Preiss R (2010). Serum interleukin-6 levels in colorectal cancer patients—a summary of published results. Int J Colorectal Dis.

[10] Milicevic N (2014). Comparison between clinical significance of serum proinflammatory protein interleukin-6 and classic tumor markers total PSA, free PSA and free/total PSA prior to prostate biopsy. Coll Antropol.

[11] Nakashima J (2000). Serum interleukin 6 as a prognostic factor in patients with prostate cancer. Clin Cancer Res.

[12] Tam L (2007). Expression levels of the JAK/STAT pathway in the transition from hormone-sensitive to hormone-refractory prostate cancer. Br J Cancer.

[13] Waldner MJ, Foersch S, Neurath MF (2012). Interleukin-6—a key regulator of colorectal cancer development. Int J Biol Sci.

[14] Rojas A (2011). IL-6 promotes prostate tumorigenesis and progression through autocrine cross-activation of IGF-IR. Oncogene.

[15] Kishimoto T (1989). The biology of interleukin-6. Blood.

[16] Yu SH (2015). A paracrine role for IL6 in prostate cancer patients: lack of production by primary or metastatic tumor cells. Cancer Immunol Res.

[17] Jones SA (2005). IL-6 transsignaling: the in vivo consequences. J Interferon Cytokine Res.

[18] McLoughlin RM (2005). IL-6 trans-signaling via STAT3 directs T cell infiltration in acute inflammation. Proc Natl Acad Sci U S A.

[19] Yu H (2014). Revisiting STAT3 signalling in cancer: new and unexpected biological functions. Nat Rev Cancer.

[20] Heinrich PC (2003). Principles of interleukin (IL)-6-type cytokine signalling and its regulation. Biochem J.

[21] Lu K (2015). The STAT3 inhibitor WP1066 reverses the resistance of chronic lymphocytic leukemia cells to histone deacetylase inhibitors induced by interleukin-6. Cancer Lett.

[22] Yang, Z., et al., Acquisition of resistance to trastuzumab in gastric cancer cells is associated with activation of IL-6/STAT3/Jagged-1/Notch positive feedback loop. Oncotarget. 2015;6(7):5072–87 10.18632/oncotarget.3241 PMC4467134 25669984

[23] Bournazou E, Bromberg J (2013). Targeting the tumor microenvironment: JAK-STAT3 signaling. JAKSTAT.

[24] Guo Y (2012). Interleukin-6 signaling pathway in targeted therapy for cancer. Cancer Treat Rev.

[25] Middleton K (2014). Interleukin-6: an angiogenic target in solid tumours. Crit Rev Oncol Hematol.

[26] Zhang K (2016). Association between interleukin-6 polymorphisms and urinary system cancer risk: evidence from a meta-analysis. Onco Targets Ther.

[27] Chen J (2016). Association between polymorphisms in selected inflammatory response genes and the risk of prostate cancer. Onco Targets Ther.

[28] Chen CH (2015). Role of interleukin-6 gene polymorphisms in the development of prostate cancer. Genet Mol Res.

[29] Coulie PG, Stevens M, Van Snick J (1989). High- and low-affinity receptors for murine interleukin 6. Distinct distribution on B and T cells. Eur J Immunol.

[30] Hammacher A (1994). Structure-function analysis of human IL-6: identification of two distinct regions that are important for receptor binding. Protein Sci.

[31] Greenberg NM (1994). The rat probasin gene promoter directs hormonally and developmentally regulated expression of a heterologous gene specifically to the prostate in transgenic mice. Mol Endocrinol.

[32] Greenberg NM (1995). Prostate cancer in a transgenic mouse. Proc Natl Acad Sci U S A.

[33] Cervantes-Arias A, Pang LY, Argyle DJ (2013). Epithelial-mesenchymal transition as a fundamental mechanism underlying the cancer phenotype. Vet Comp Oncol.

[34] Fawcett J, Harris AL (1992). Cell adhesion molecules and cancer. Curr Opin Oncol.

[35] Abdulkadir SA (2002). Conditional loss of Nkx3.1 in adult mice induces prostatic intraepithelial neoplasia. Mol Cell Biol.

[36] Kim MJ (2002). Nkx3.1 mutant mice recapitulate early stages of prostate carcinogenesis. Cancer Res.

[37] Schweizer MT, Yu EY (2015). Persistent androgen receptor addiction in castration-resistant prostate cancer. J Hematol Oncol.

[38] Garbers C, Aparicio-Siegmund S, Rose-John S (2015). The IL-6/gp130/STAT3 signaling axis: recent advances towards specific inhibition. Curr Opin Immunol.

[39] Taniguchi K, Karin M (2014). IL-6 and related cytokines as the critical lynchpins between inflammation and cancer. Semin Immunol.

[40] Candido J, Hagemann T (2013). Cancer-related inflammation. J Clin Immunol.

[41] Guven Maiorov E (2013). The structural network of inflammation and cancer: merits and challenges. Semin Cancer Biol.

[42] Janakiram NB, Rao CV (2014). The role of inflammation in colon cancer. Adv Exp Med Biol.

[43] Taverna, G., et al., Inflammation and prostate cancer: friends or foe? Inflamm Res, 2015 10.1007/s00011-015-0812-2 25788425

[44] Aggarwal BB (2006). Inflammation and cancer: how hot is the link?. Biochem Pharmacol.

[45] Balkwill F, Charles KA, Mantovani A (2005). Smoldering and polarized inflammation in the initiation and promotion of malignant disease. Cancer Cell.

[46] Colotta F (2009). Cancer-related inflammation, the seventh hallmark of cancer: links to genetic instability. Carcinogenesis.

[47] Zarogoulidis P (2013). Interleukin-6 cytokine: a multifunctional glycoprotein for cancer. Immunome Res.

[48] Kubo M, Hanada T, Yoshimura A (2003). Suppressors of cytokine signaling and immunity. Nat Immunol.

[49] Murakami M, Hirano T (2012). The pathological and physiological roles of IL-6 amplifier activation. Int J Biol Sci.

[50] Isomoto H (2009). Epigenetic alterations in cholangiocarcinoma-sustained IL-6/STAT3 signaling in cholangio-carcinoma due to SOCS3 epigenetic silencing. Digestion.

[51] Pierconti F (2011). Epigenetic silencing of SOCS3 identifies a subset of prostate cancer with an aggressive behavior. Prostate.

[52] Cutolo M, Paolino S, Pizzorni C (2014). Possible contribution of chronic inflammation in the induction of cancer in rheumatic diseases. Clin Exp Rheumatol.

[53] Shanmugam MK, Sethi G (2013). Role of epigenetics in inflammation-associated diseases. Subcell Biochem.

[54] Tilg H, Moschen AR (2006). Adipocytokines: mediators linking adipose tissue, inflammation and immunity. Nat Rev Immunol.

[55] Jayachandran A, Dhungel B, Steel JC (2016). Epithelial-to-mesenchymal plasticity of cancer stem cells: therapeutic targets in hepatocellular carcinoma. J Hematol Oncol.

[56] Li X (2016). Lung tumor exosomes induce a pro-inflammatory phenotype in mesenchymal stem cells via NFkappaB-TLR signaling pathway. J Hematol Oncol.

[57] Yin X (2015). Coexpression of gene Oct4 and Nanog initiates stem cell characteristics in hepatocellular carcinoma and promotes epithelial-mesenchymal transition through activation of Stat3/Snail signaling. J Hematol Oncol.

[58] MacLennan GT (2006). The influence of chronic inflammation in prostatic carcinogenesis: a 5-year followup study. J Urol.

[59] Nakai Y, Nonomura N (2013). Inflammation and prostate carcinogenesis. Int J Urol.

[60] Sfanos KS, Hempel HA, De Marzo AM (2014). The role of inflammation in prostate cancer. Adv Exp Med Biol.

