# Supplementary material for: Prostate-specific IL-6 transgene autonomously induce prostate neoplasm through amplifying inflammation in the prostate and peri-prostatic adipose tissue
Source: J Hematol Oncol. 2017 Jan 11;10:14. doi: 10.1186/s13045-016-0386-7 (PMC5225646; doi:10.1186/s13045-016-0386-7)
Supplement: Additional file 1: Figure S1. — Representative histology of a 103-week-old mouse (#1825) that developed poorly differentiated tumor in the prostate. CD45 stains for all leukocyte antigens in the infiltrates. Supplement Figure 2. Exogenous human IL-6 induces paracrine mouse IL-6 expression in two mouse prostate tumor cell lines, the TRAMP-C2 and MyC-Cap. Fifty nanogram per milliliter of human IL-6 was supplemented in the culture media. Cells were harvested at 24 and 48 hr of culture. Relative mouse IL-6 expression was assessed by quantitative RT-PCR. Table S1. Function of the most significantly upregulated genes with more than log2 fold changes in the volcano plot. Table S2. Upregulated inflammation-associated genes in the prostate of pbIL-6 mice that are not highlighted on the volcano plot. Table S3. Upregulated oncogenic genes in the prostate of pbIL-6 mice that are not highlighted on the volcano plot. Table S4. Upregulated metabolism-associated genes in the prostate of pbIL-6 mice that are not highlighted on the volcano plot. Table S5. List of primers used for qRT-PCR. (PDF 329 kb) [file 13045_2016_386_MOESM1_ESM.pdf]

Figure S1

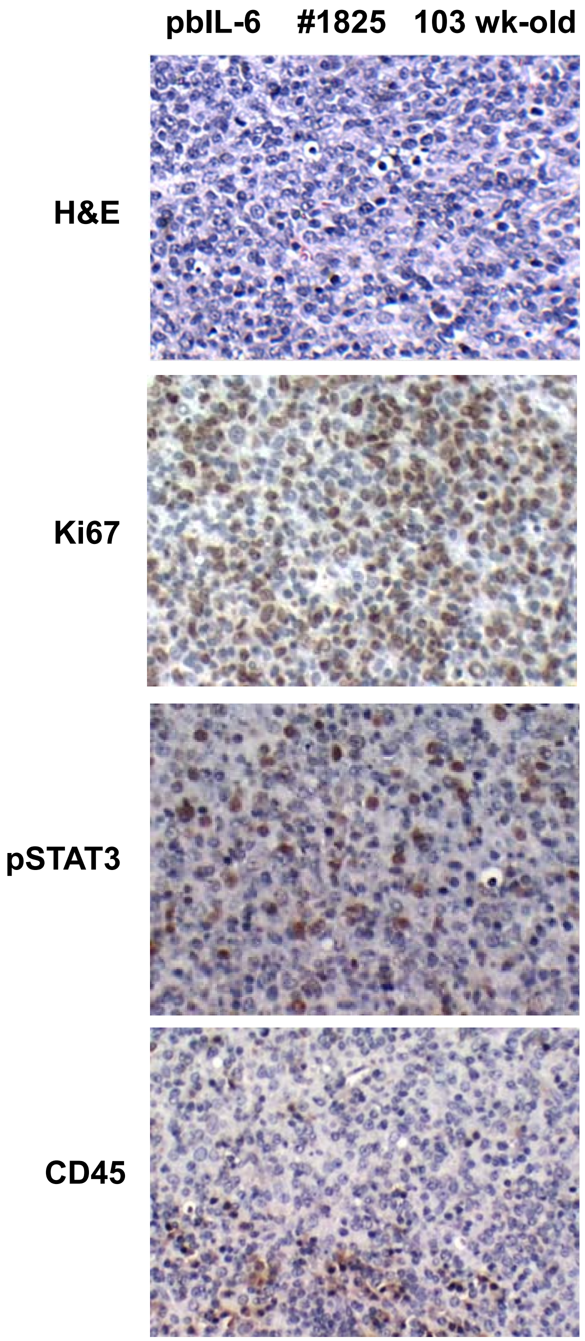

Figure S1. Representative histology of a 103 wk-old mouse (#1825) that development poorly differentiated tumor in the prostate. CD45 stains for all leukocyte antigens in the infiltrates.

Figure S2

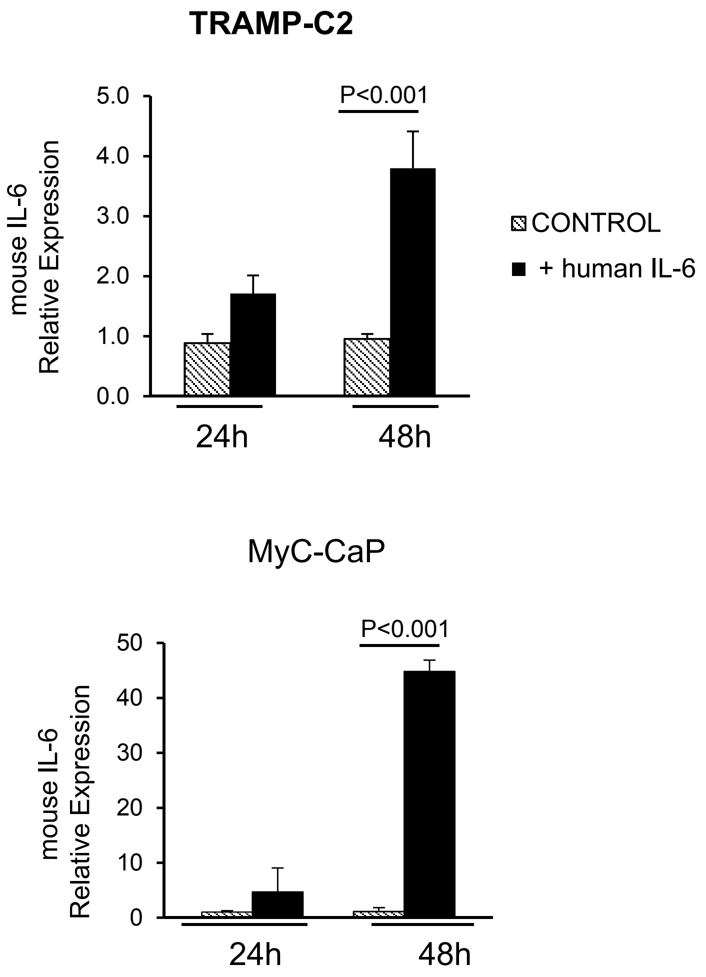

Supplement Figure 2. Exogenous human IL-6 induces paracrine mouse IL-6 Expression in two mouse prostate tumor cells lines, the TRAMP-C2 and MyC-Cap. 50 ng/ml of human IL-6 was supplemented in the culture media. Cells were harvested at 24 hr and 48 hr of culture. Relative mouse IL-6 expression was assessed by quantitative RT-PCR.

Table S1. Function of the most significantly upregulated genes with more than Log2 fold changes in the Volcano plot

Gene ID	encoding protein	function
**up-regulated**		
BCL3	B cell leukemia/lymphoma 3 protein	proto-oncogene, pro-survival and pro-inflammation
C1R	Complelement 1r	complement system, pro-inflammation
CSGALNACT1	Chondroitin Sulfate N-Acetylgalactosaminyltransferase 1	pro-oncogenic, promote wnt/β-catenin pathway
CXCL6	chemokine ligand 6	pro-inflammation
CYP1B1	Cytochrome P450 1B1	Steriod Metabolism and fat production
IER3	Immediate early response 3	regulates cycle progression and pro-ongogenic
IL-27Ra	Interleukin 27 receptor	pro-inflammation
IL-6	Interleukin 6	Pro-inflammation
LRG1	Leucine-rich α2 glycoprotein 1	inflammatory protein, associated with malignancy
MAFF	v-maf avian musculoaponeurotic fibrosarcoma oncogene homolog F	Pro-oncogene, Transcriptional factor, stress response
NFKB1a	Nuclear factor NF-kappa-B p105 subunit	Transcriptional factor, Pro-inflammation
TGF-β1	transforming growth factor beta superfamily of cytokines	pro-oncogenic and pro-inflammation
**down-regulated**		
EDIL-3	EGF-like repeats and Discoidin I-Like Domains 3	Extracellular matrix, ligand for intergrins
EMP2	Epithelial membrane protein-2	regulates angionenesis
NPTX1	neuronal pentraxin 1	epigenetic regulator, methylation regulator

Table S2. Upregulated inflammation-associated Genes in the prostate of pbIL-6 mice that are not highlighted on the Volcano Plot

C1S	complement component 1, s subcomponent
CASP1	caspase 1, apoptosis-related cysteine peptidase
CASP10	caspase 10, apoptosis-related cysteine peptidase
CASP4	caspase 4, apoptosis-related cysteine peptidase
CFB	complement factor B
CSF	Colony stimulating factor 1 (macrophage)
CTAGE1	cutaneous T-cell lymphoma-associated antigen 1
CTSS	cathepsin S
CTSZ	cathepsin Z
CXCL1	chemokine (C-X-C motif) ligand 1
CXCL2	chemokine (C-X-C motif) ligand 2
CXCL3	chemokine (C-X-C motif) ligand 3
G0S2	lymphocytes G0/G1switch 2
GCNT2	glucosaminyl (N-acetyl) transferase 2, I-branching enzyme
ICAM1 (CD54)	intercellular adhesion molecule 1, binds to CD11a/CD18)
IFI16	interferon, gamma-inducible protein 16
IFI44L	interferon-induced protein 44-like
IL4R	interleukin 4 receptor
IL7R	interleukin 7 receptor
IL8	interleukin 8
INHBA	inhibin, beta A
ISG20	interferon stimulated exonuclease gene 20kDa
ITGA2	integrin, alpha 2 (CD49B, alpha 2 subunit of VLA-2 receptor)
ITGB2 (CD18)	integrin, beta 2 (CD18)
KLF4	Kruppel-like factor 4
NAIP	NLR family, apoptosis inhibitory protein
NFATC1	nuclear factor of activated T-cells, cytoplasmic, calcineurin-dependent 1
NLRP1	NLR family, pyrin domain containing 1
NNMT	nicotinamide N-methyltransferase
PTX3	pentraxin-related gene, rapidly induced by IL-1 beta
PVRL3	poliovirus receptor-related 3
RFTN1	raftlin, lipid raft linker 1
S100A3	S100 calcium binding protein A3
S100A9	S100 calcium binding protein A9
SAA1	serum amyloid A1
SAA2	serum amyloid A2
SOCS3	suppressor of cytokine signaling 3
TLR2	toll-like receptor 2
TNFAIP3	tumor necrosis factor, alpha-induced protein 3
TRBV5-4	T cell receptor beta variable 5-4
VEGFC	vascular endothelial growth factor C
VNN1	vanin 1

Table S3. Upregulated oncogenic genes in the prostate of pbIL-6 mice that are not highlighted on the Volcano Plot

CASC5	cancer susceptibility candidate 5
CCND1	cyclin D1
CDC2L1	cell division cycle 2-like 1 (PITSLRE proteins)
CDH2	cadherin 2, type 1, N-cadherin (neuronal)
COL16A1	collagen, type XVI, alpha 1
CTNND1	catenin (cadherin-associated protein), delta 1
FOSL1	FOS-like antigen 1
IFNGR1	interferon gamma receptor 1
IGFI	insulin-like growth factor 1
JUN	jun oncogene
KLF4	Kruppel-like factor 4
LCN2	lipocalin 2
MUC1	mucin 1, cell surface associated
OSMR	oncostatin M receptor
PBX2	pre-B-cell leukemia homeobox 2
PMAIP1	phorbol-12-myristate-13-acetate-induced protein 1
RAB27B	RAB27B, member RAS oncogene family
RABL4	RAB, member of RAS oncogene family-like 4
RASSF4	Ras association (RalGDS/AF-6) domain family member 4
RELB	v-rel reticuloendotheliosis viral oncogene homolog B
RHEBL1	Ras homolog enriched in brain like 1
SERPINB3	serpin peptidase inhibitor, clade B, member 3
SERPINB4	serpin peptidase inhibitor, clade B, member 4
SERPINB8	serpin peptidase inhibitor, clade B, member 8
SERPINE2	serpin peptidase inhibitor, clade E, member 2
TMPRSS2	Androgen regulated transmembrane protease, serine 2

Table S4. Upregulated metabolism-associated genes in the prostate of pbIL-6 mice that are not highlighted on the Volcano Plot

APOL1	apolipoprotein L, 1
GPRC5B	G protein-coupled receptor, may be associated with insulin secretion
GLS	glutaminase
KMO	kynurenine 3-monooxygenase (kynurenine 3-hydroxylase)
LOXL2	lysyl oxidase-like 2
PLAT	plasminogen activator, tissue
SAT1	spermidine/spermine N1-acetyltransferase 1
SCD	stearoyl-CoA desaturase (delta-9-desaturase)
XAF1	XIAP associated factor 1

Table S5. List of primers used for qRT-PCR.

Gene (mouse)	Forward	Reverse
E-Cadherin	CGAGAGAGTTACCCTACATA	GTGTTGGGGGCATCATCATC
N-cadherin	AGCGCAGTCTTACCGAAGG	TCGCTGCTTTCATACTGAACTTT
Vimentin	CGGCTGCGAGAGAAATTGC	CCACTTTCCGTTCAAGGTCAAG
IGF-I	TCATGTCGTCTTCACACCTCTTCT	CCACACACGAACTGAAGAGCAT
IGF-II	ACAACTTCGATTTGAACCACATTC	GAGAGCTCAAACCATGCAAACT
AR	TACTTTCCACCCCAGAAGACCTGC	CATTTCCGAAGACGACAAGATGG
mIL-6	CAAAGCCAGAGTCCTTCAGA	GATGGTCTTGGTCCTTAGCC
hIL-6	GAACTCCTTCTCCACAAGCG	GAATCCAGAGATTGGAAGCATCC
gp130	CCAGCAACGAGGAGAATGAGT	TGTGCACCACAGTGGAGTACTG
c-Fos	GACTTCTTGTTTCCGGCATCA	AGGTCCACATCTGGCACAGAG
K-Ras	GCTCAGGAGTTAGCAAGGAG	GTATTCACATAACTGTACACCTTG
GAPDH	GACAACTTTGGCATTGTGG	ATGCAGGGATGATGTTCTG
